# Plant Unsaturated Fatty Acids: Biosynthesis and Regulation

**DOI:** 10.3389/fpls.2020.00390

**Published:** 2020-04-23

**Authors:** Mei He, Chun-Xue Qin, Xu Wang, Nai-Zheng Ding

**Affiliations:** College of Life Science, Shandong Normal University, Jinan, China

**Keywords:** unsaturated fatty acids, biosynthesis, regulation, transcriptional factors, signaling pathways

## Abstract

In most plants, major unsaturated fatty acids (UFAs) are three C18 species, namely, oleic (18:1), linoleic (18:2), and α-linolenic (18:3) acids. These simple compounds play multiple crucial roles *in planta* and are also important economic traits of oil crops. The enzymatic steps of C18 UFA biosynthesis have been well established. However, the associated FA/lipid trafficking between the plastid and the endoplasmic reticulum remains largely unclear, as does the regulation of the expression and activities of the involved enzymes. In this review, we will revisit the biosynthesis of C18 UFAs with an emphasis on the trafficking, and present an overview of the key enzymes and their regulation. Of particular interest is the emerging regulatory network composed of transcriptional factors and upstream signaling pathways. The review thereby provides the promise of using physical, biochemical and/or genetic means to manipulate FA composition and increase oil yield in crop improvement.

## Introduction

Unsaturated fatty acids (UFAs), alphatic carboxylic acids with one or more double bonds mostly in *cis* configuration, are fundamental to higher organisms. In most plants, the predominant UFAs are three 18-carbon (C18) species, i.e., 18:1 (oleate), 18:2 (linoleate), and 18:3 (α-linolenate) (Harwood, 1988), where m:n stands for an FA with m carbon atoms and n double bonds. These simple compounds play multiple crucial roles and are deeply associated with both abiotic and biotic stresses. Besides membrane ingredients and modulators in glycerolipids, as well as carbon and energy reserve in triacylglycerols (TAGs), C18 UFAs serve as intrinsic antioxidants, precursors of various bioactive molecules [typically the stress hormone jasmonic acid (JA)], and stocks of extracellular barrier constituents such as cutin and suberin (Ohlrogge and Browse, 1995; Harwood, 1996; He et al., 2018). Moreover, C18 UFAs *per se* also play regulatory roles in plant defense (Lim et al., 2017). 18:1, for example, is involved in the crosstalk between salicylic acid (SA) and JA signaling pathways against pathogen invasion (Kachroo et al., 2001).

C18 UFAs are also important economic traits of oil crops. For one thing, 18:2 and 18:3, the two polyunsaturated FAs (PUFAs), are dietary essential FAs, because we human beings are incapable of their biosynthesis; however, high 18:1 or 18:3 and low 18:2 are beneficial for our health. For another, these organic substances are raw materials of manifold commodities such as biofuels, cosmetics, detergents, and pharmaceuticals (Harwood, 1996). Of note, their anti-stress roles, wholesome properties and industrial applications all highlight the significance of manipulating FA composition and increasing oil yield in crop improvement. In this review, therefore, we will revisit the biosynthesis of C18 UFAs and present an overview of the key enzymes and the regulation of their expression and activities. An emphasis is put on the associated FA/lipid trafficking between the plastid and the endoplasmic reticulum (ER). Of particular interest is the regulatory network composed of transcriptional factors (TFs) and upstream signaling pathways, which is beginning to be deciphered.

## Biosynthesis of C18 Unsaturated Fatty Acids

**FIGURE 1:**
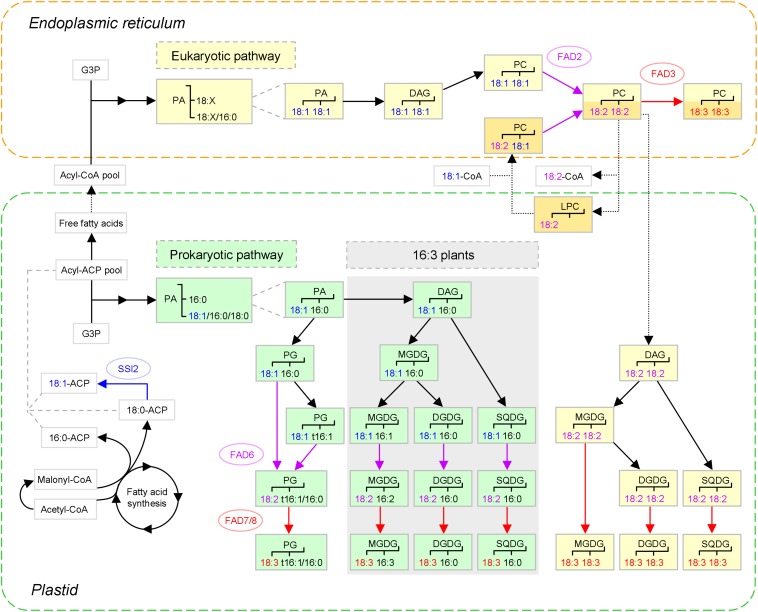
The major biosynthetic pathways of plant C18 unsaturated fatty acids (UFAs) (based on Ohlrogge and Browse, 1995; Karki et al., 2019). After 18:1 is synthesized *de novo* in plastids, the generation of 18:2 and 18:3 is coupled to that of membrane glycerolipids, which is conducted in two pathways: the ‘prokaryotic pathway’ in plastids, and the ‘eukaryotic pathway’ in the endoplasmic reticulum (ER), as marked in green and yellow, respectively. To illustrate the acyl editing way of 18:1 incorporation (distinguished from the *de novo* way with darker color), PC(18:2, 18:2) is used as an example substrate. Nascent FAs are largely channeled to the acyl editing way, whereas those exchanged instead enter the *de novo* way of eukaryotic glycolipid synthesis. The three C18 UFAs and the corresponding fatty acid desaturases (FADs) and reactions are shown in different colors. The dotted arrows denote that trafficking between the plastid and the ER is involved. Prokaryotic glycolipid synthesis is only present in ‘16:3 plants’ characterized by MGDG(18:3, 16:3), as indicated by shading. PA and PG of likely ER origin are not shown. SSI2, SUPPRESSOR OF SA INSENSITIVE 2; t16:1, *trans*-16:1; 18:X, C18 FAs; G3P, glycerol-3-phosphate; PA, phosphatidic acid; DAG, diacylglycerol; PG, phosphatidylglycerol; MGDG, monogalactosyldiacylglycerol; DGDG, digalactosyldiacylglycerol; SQDG, sulfoquinovosyldiacylglycerol; PC, phosphatidylcholine; LPC, lysoPC.

The enzymatic cascade for C18 UFA generation has been well established and documented (e.g., reviews Ohlrogge and Browse, 1995; Harwood, 1996), as illustrated in Figure 1. Here, the biosynthetic procedure in *Arabidopsis* is taken as an example. Briefly, in plastids, FAs are synthesized *de novo* from acetyl-coenzyme A (CoA), owing to the concerted action of acetyl-CoA carboxylase (ACC) and FA synthase (FAS). Once produced, 18:0 conjugated to acyl carrier protein (ACP) primarily enters the unsaturation program administered by a series of FA desaturases (FADs). 18:1-ACP is rapidly created by stearoyl-ACP desaturase (SAD). However, the biosynthesis of C18 PUFAs is coupled to that of membrane glycerolipids, which is conducted in two parallel pathways — the ‘prokaryotic’ one in plastids and the ‘eukaryotic’ one in the ER.

To form a glycerolipid, two acyl chains are successively esterified to the *sn*-1 and *sn*-2 positions of glycerol-3-phosphate (G3P). In the prokaryotic pathway, 18:1 will only appear at *sn*-1 depending on the substrate preference of plastidial G3P acyltransferase (GPAT), as *sn*-2 is almost exclusively provided with 16:0. After vector glycerolipids such as phosphatidylglycerol (PG) and monogalactosyldiacylglycerol (MGDG) are generated, 18:1(Δ9) is processed to 18:2(Δ9, 12) by ω-6 FAD6 and then to 18:3(Δ9, 12, 15) by ω-3 FAD7 or FAD8, where Δ and ω are counting from the carboxylic and methyl ends, respectively (Ohlrogge and Browse, 1995; Harwood, 1996).

In the eukaryotic pathway, acyl chains need to be firstly exported to the ER (for review, see Li et al., 2016; LaBrant et al., 2018). During the course, each is hydrolyzed by acyl-ACP thioesterase (FAT) into free FA and then re-activated by long-chain acyl-CoA synthetase (LACS). However, it is unknown yet which of the nine LACSs are involved (Jessen et al., 2015). Although free FAs may transit the plastid inner envelope membrane (IEM) via simple diffusion, FATTY ACID EXPORT 1 (FAX1) — an integral protein embedded in the IEM via α-helical membrane-spanning domains — emerged as a likely transporter. Levels of ER-derived lipids decreased upon *FAX1* mutation but increased upon *FAX1* overexpression (Li et al., 2015). The novel FAX family consists of seven members. While FAX1 plays a prominent role in leaves and flowers (Li et al., 2015), FAX2 and FAX4 are important for seed oil accumulation (Tian et al., 2019; Li et al., 2020). Of note, FAX1 can rescue yeast (*Saccharomyces cerevisiae*) mutant defective in FA transport with a cargo preference for 16:0 over 18:1; however, 18:1 is the major FA exported out of plastids (Li et al., 2015). This might be due to the high availability of 18:1, an 18:1-favoring family member, and/or other export mechanisms.

Currently, how free FAs cross the intermembrane space and then the outer envelope membrane (OEM) of plastids remain questions to be answered. One candidate is the OEM-localized LACS9 that might drive FA export via vectorial acylation, a coupled process of transport and activation (Li et al., 2016). However, as with the ER-bound LACS4, its known role so far is to work for the reverse, i.e., ER-to-plastid lipid transfer. This is manifested by the *lacs4/9* double mutant showing impaired incorporation of radiolabeled FAs into plastidial galactolipids of ER origin, but not ER phospholipids. Moreover, LACS8 may play an overlapping role in the process, as suggested by the lethality of the triple mutant (Jessen et al., 2015).

In the cytoplasm, acyl-CoA binding proteins (ACBPs) (Li et al., 2016) offer an interorganelle delivery route for FAs to reach the cytosolic face of the ER where phosphatidylcholine (PC) synthesis occurs (Botella et al., 2017). ACBP4, -5, and -6 are cytosolic and may play overlapping yet distinct roles in seed acyl-lipid metabolism. Particularly, 18:1-CoA accumulates in both embryos and seedlings of the *acbp6* mutant, suggesting the involvement of ACBP6 (Hsiao et al., 2014). In addition, acylcarnitines were supposed to participate in shuffling FAs from plastids to the ER, based on the following facts: (1) acylcarnitine is a form of FA trafficking in animals and yeasts; (2) acylcarnitines are present in plants, with 18:1-carnitine being the relatively abundant one; (3) chloroplasts harbor carnitine transferase activities; (4) acylcarnitine pool enrichment is concomitant to membrane lipid biosynthesis (see review Jacques et al., 2018).

There are two ways for 18:1 to be incorporated into PC for desaturation (for review, see LaBrant et al., 2018). One is the *de novo* way of PC synthesis, in which 18:1 may take both positions of G3P, especially *sn*-2, which is biased toward it. Subsequently, phosphatidic acid (PA) is modified via diacylglycerol (DAG) into PC (Ohlrogge and Browse, 1995). The other is the acyl editing way of PC turnover, in which 18:1 can directly enter PC, mostly at *sn*-2 (Bates et al., 2009). This is based on the dynamic interconversion between PC and lysoPC. Notably, lysoPC acyltransferase (LPCAT) is capable of catalyzing both forward and reverse reactions and thus fulfilling the cycle on its own (Wang L. et al., 2012). It appears that nascent 18:1 exported out of plastids favors the shortcut in most tissues (LaBrant et al., 2018), which is enabled by the plastid-associated LPCAT1 and -2 (Wang L. et al., 2012; Karki et al., 2019). Next, the recycled PC is transported to the ER, possibly via ER-plastid membrane contact sites (MCSs). ACBPs might also be engaged, given their ability to bind PC (Li et al., 2016). Such renders an alternative plastid-to-ER delivery route for FAs, with PC serving as a carrier.

The unsaturation program is then carried on by ω-6 FAD2 and ω-3 FAD3. When 18:2-containing PC, e.g., PC(18:2, 18:2), is generated, lipid trafficking from the ER back to plastids occurs to supply its DAG backbone for glycolipid production (Ohlrogge and Browse, 1995). To date, the lipid species transferred and the underlying mechanisms are still unclear. Candidates for ER-to-OEM transport comprise PC and its metabolites — lysoPC, PA and DAG (LaBrant et al., 2018). In fact, the OEM outer leaflet contains a substantial amount of PC. ALA10, a phospholipid flippase of the P4-type ATPase family, was speculated to enrich PC in ER-plastid MCSs for translocation, given that its overexpression raised the level of 18:2-containing PC in chloroplasts (see review Botella et al., 2017).

Besides ACBPs or other lipid transfer proteins for direct shuttle, PC might turn to lysoPC that can rapidly move from the ER to chloroplasts (Bessoule et al., 1995). Based on the involvement of LACS4/9 in the process and a specific accumulation of free 18:2 in the double mutant, Jessen et al. (2015) proposed a model as follows. The transfer is initiated by an ER phospholipase A_2_ (PLA_2_) that prefers PC with *sn*-2 18:2. While the resultant lysoPC migrates to the plastid envelope, the liberated 18:2 is activated by LACS4/9 to establish a local 18:2-CoA pool, so that it can be specifically used by a plastidial LPCAT to regenerate PC. Of note, such coupled transport avoids the issue of losing *sn*-2 C18 FAs indicative of the ER origin, but is against the positional bias observed in leek (*Allium porrum*) upon stereospecific labeling — *sn*-2 in PC vs. *sn*-1 in MGDG (Mongrand et al., 2000), unless a pool of *sn*-1 labeled PC is specialized as the MGDG precursor (like PC3 below).

Now, the lysoPC-mediated way is challenged by the new finding that LPCAT1 and -2 are required for acyl flux to, but not from, the ER (Karki et al., 2019). This was revealed by *in vivo* labeling analyses conducted on the *act1 lpcat1 lpcat2* triple mutant, taking advantage of the *act1* background that eliminates the prokaryotic pathway of the glycolipid assembly and elevates the eukaryotic pathway for compensation. It turned out that the direct incorporation of nascent FAs into PC was impaired. However, acyl flux was shifted toward *de novo* PC synthesis with a higher *sn*-1 label and there was little effect on MGDG production. Accordingly, Karki et al. (2019) put forward spatial organization of metabolically distinct pools of PC, such as PC1 for acyl editing, PC2 as the bulk one from *de novo* synthesis, and PC3 (derived from PC2) for glycolipid formation. Possibly, PC1 and PC3 are allocated to different MCSs.

In their model, the neosynthesized FAs are largely channeled by LPCAT into PC for further desaturation, whereas the exchanged ones (cleaved by PLA_2_ or LPCAT) instead enter the *de novo* eukaryotic pathway. Interestingly, a support is offered by an *in vivo* lipid ‘tag and track’ approach established via introducing an ER-resident Δ6 FAD that specifically acts on the *sn*-2 acyl chain of PC (Hurlock et al., 2018). A notable outcome was that ∼10% of the unusual Δ6 FAs, e.g., 18:4(Δ6, 9, 12, 15), in PC switched to *sn*-1, suggesting that they were released via acyl editing and then reused for *de novo* synthesis. Then, considering the reduced seed oil yield in *lacs4/9* (Jessen et al., 2015), it is possible that the two LACSs are involved in re-activating PLA_2_-freed FAs, thereby affecting both TAG and eukaryotic galactolipid synthesis. This can be clarified by checking whether the *de novo* way is impaired in *lacs4/9*.

Notably, there is another way for lysoPC to regenerate PC, enabled by lysoPC transacylase (LPCT) and glycerophosphocholine acyltransferase (GPCAT) acting in concert. LPCT catalyzes acyl transfer from one lysoPC to another, resulting in PC and GPC. GPCAT then converts GPC back to lysoPC by using acyl-CoA (Lager et al., 2015). If this way operates in the OEM, then LACS4/9 can have an effect on lysoPC recycling. However, unless 16:0-containing PC is excluded from the transport and 16:0-CoA is unavailable for GPCAT, the C18 criterion will be challenged, as 16:0 may be transferred to *sn*-2, although 16:0-lysoPC is a preferred acyl acceptor (Lager et al., 2015). Moreover, a specific pool or compartmentalization might also be required, otherwise it would be incompatible with the observation that there is little change in the stereospecificity of the Δ6 FAs in chloroplastic PC (still ∼90% at *sn*-2) (Hurlock et al., 2018).

With regard to plastid import, PA and DAG are candidates. Plastid envelope membranes harbor a lipid importing machinery called TGD for abnormal accumulation of trigalactosyldiacylglycerol (TGDG) in the mutants. It is composed of five proteins, with TGD4 forming a β-barrel in the OEM, TGD1-3 assembling a bacterial-type ABC transporter complex in the IEM, and TGD5 being a likely bridge in between (see reviews Li et al., 2016; Hölzl and Dörmann, 2019). PA is proposed to be a cargo of TGD, since both TGD4 and TGD2 can specifically bind it (Lu and Benning, 2009; Wang Z. et al., 2012). However, there is a possibility that PA is a functional component of TGD, acting as a membrane destabilizer to reduce the energy barrier for transport (LaBrant et al., 2018). Of note, the presence of ER-derived acyl chains in chloroplastic PG was tracked by using the Δ6 tag (Hurlock et al., 2018). This might result from PA import, because PA can be converted to not only DAG but PG in plastids (Figure 1), or PA generation from the imported DAG.

Surprisingly, a great discrepancy existed in the distribution of the Δ6 tag in the chloroplastic glycerolipid species examined — ∼90% in PC, ∼30% in PA, and ∼50% in the four thylakoid lipids occupied *sn*-2, rendering the precursor-product relationship elusive. This observation, together with the coexistence of eukaryotic 18:4 and prokaryotic 16:3 in the same MGDG molecule, led to the assumption that extensive acyl editing occurred in thylakoid lipids, which might be triggered by the presence of unusual acyl groups (Hurlock et al., 2018). However, if a proportion of the imported PA undergoes *sn*-2 deacylation and thus the characteristic 16:0 acylation, the phenomena can be accounted for. Interestingly, 16:3 might also have a eukaryotic origin, provided that the LPCT-GPCAT way is involved. Perhaps, tracking the Δ6 tag in the *lpcat1/2* background coupled with PLA_2_ inhibition would give an answer. Finally, once DAG is modified to glycolipids, 18:2 can be further desaturated to 18:3 by FAD7/8.

## Key Fatty Acid Synthetic Enzymes and Their Regulation

**FIGURE 2:**
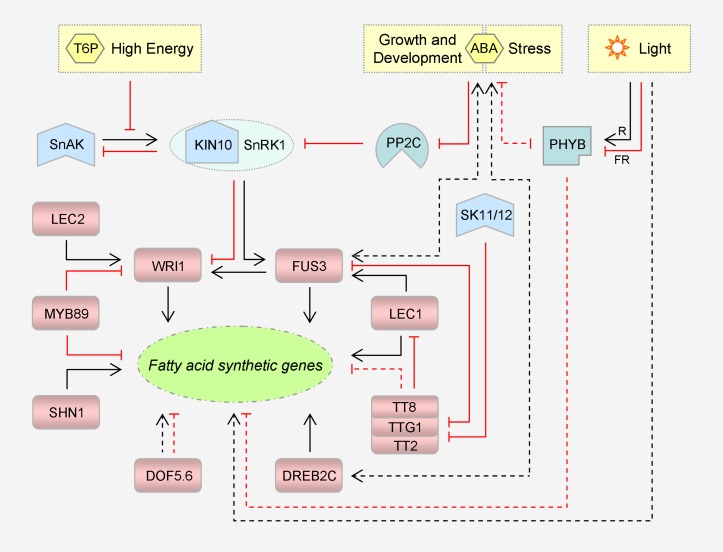
Representative transcriptional factors and signaling pathways that regulate plant fatty acid synthesis. Please note that these transcriptional factors target different sets of fatty acid synthetic genes, directly or indirectly. TTG1, TT2 and TT8 can form a ternary complex to modulate gene expression. KIN10 is a catalytic subunit of SnRK1. Inhibitory effects are marked in red. Dashed lines denote indirect regulation. For simplicity, the intricate interactions among LEC1, LEC2 and FUS3 are not shown. T6P, trehalose 6-phosphate; SnRK1, sucrose non-fermenting 1-related protein kinase 1; SnAK: SnRK1-activating kinase; ABA, abscisic acid; PP2C, protein phosphatase 2C; R, red light; FR, far-red light; PHYB, phytochrome B; SK11/12, shaggy-like kinases 11/12; DOF, DNA binding with one finger; DREB, dehydration-responsive element-binding; WRI1, WRINKLED1; FUS3, FUSCA3; LEC1/2, LEAFY COTYLEDON 1/2; SHN1, SHINE1; TTG1, TRANSPARENT TESTA GLABRA 1; TT2/8, TRANSPARENT TESTA 2/8.

FA biosynthesis is a basic yet dynamic process that is developmentally and physiologically regulated. Great strides have been made in characterizing the key enzymes for FA synthesis and understanding the regulation of their expression and activities. Particularly, ACC, which catalyzes the committed and rate-limiting step of *de novo* FA synthesis, has attracted much attention, leading to the discoveries of a large variety of genetic and biochemical mechanisms underlying its regulation, as described below. Given that most TFs identified (examples shown in Figure 2) target more than one FA synthetic gene, transcriptional regulation will be addressed in a separate section. Moreover, there is a large variation in the gene number of each enzyme in the plant kingdom; gene information of *Arabidopsis* will be introduced for reference.

### Key Enzymes and Non-transcriptional Regulation

#### Acetyl-CoA Carboxylase

In nature, ACC exists in two physically distinct types: the prokaryotic one as a heteromeric multisubunit complex (htACC), and the eukaryotic one as a homomeric multidomain polypeptide (hmACC). In either form, ACC activity is based on four components — biotin caboxyl carrier protein (BCCP), biotin carboxylase (BC), and the α- and β-subunit of carboxyltransferase (CT). The majority of plants require both types, localizing htACC in plastids and hmACC in the cytoplasm (see reviews Sasaki and Nagano, 2004; Salie and Thelen, 2016).

In *Arabidopsis*, of the four individual proteins that assemble htACC in the stroma, BCCP is encoded by *CAC1A/BCCP1* and *CAC1B*/*BCCP2*, while BC, α-CT and β-CT are encoded by *CAC2*, *CAC3* and *accD*, respectively. Of note, *accD* is the only FA-related gene that resides mostly in the plastome. The exceptions comprise several seed-producing species, such as Poaceae (Konishi and Sasaki, 1994) due to gene loss or Gnetophyta (Sudianto and Chaw, 2019) due to nuclear transfer. Intriguingly, in some cases, an additional copy of the nucleus-encoded hmACC has somehow acquired a plastid-targeting transit peptide, thereby appearing in plastids as well. Therein, for htACC, it acts as a replacement in Poaceae (Konishi et al., 1996) or a concomitant in some Brassicaceae members (Rousseau-Gueutin et al., 2013). Nevertheless, unlike *ACC1*, this *ACC2* gene seems to be dispensable in *Arabidopsis* (Babiychuk et al., 2011).

As the gatekeeper that governs carbon flux into FA synthesis, htACC has both its quantity and activity under tight control, which involves diverse mechanisms yet to be fully understood (Salie and Thelen, 2016). Particularly, given that the genome copy number of plastids is several hundred times higher than that of the nucleus in a cell, for efficient assembly the expression of the plastid-encoded β-CT ought to be coordinated with that of the other three nuclear-encoded subunits. This can be partly achieved at the levels post transcription, possibly via protein degradation of excess subunits (Sasaki and Nagano, 2004). β-CT appears to be a quantity control factor — overexpression of β-CT (Madoka et al., 2002), but not BCCP2 (Thelen and Ohlrogge, 2002) or BC (Shintani et al., 1997), could lead to a significant rise in protein levels of all other three subunits, though their mRNA levels were not affected.

Within the CT subcomplex, β-CT also serves as an activity control factor. In several angiosperms including pea (*Pisum sativum*) and *Arabidopsis*, RNA editing of the *accD* transcripts is required to generate a Ser-to-Leu conversion for the active form of CT (Sasaki et al., 2001). In pea leaves, β-CT is also a candidate for Ser phosphorylation, which was suggested to have a positive effect on CT activity (Savage and Ohlrogge, 1999). Moreover, upon assembly, β-CT, together with α-CT, creates a target for redox regulation. An intermolecular disulfide bond (S-S) formed between their Cys residues appears to deactivate CT, rendering reductive thioredoxin an activator, as it can break S-S into thiols with the electrons generated in photosynthesis (Sasaki et al., 1997; Kozaki et al., 2001).

Within the BC-BCCP subcomplex, BCCP is an activity control factor. To date, two regulatory proteins that work via direct interaction with BCCP have been identified. One is a family of biotin attachment domain-containing (BADC) proteins that are non-biotinylated and thus inactive analogs of BCCP (Salie et al., 2016). Surprisingly, a discrepancy has arisen regarding the role of BADCs in regulating ACC activity, namely, inhibitors by displacing BCCP (Salie et al., 2016; Keereetaweep et al., 2018) vs. activators by facilitating the assembly of BCCP-BADC-BC subcomplex (Shivaiah et al., 2020). The other is PII, a conserved sensor of the intracellular status of carbon, nitrogen and energy. It blocks BCCP via binding to the biotinylated region (Gerhardt et al., 2015), which can be reversed by 2-oxoglutarate, oxaloacetate and pyruvate (Feria Bourrellier et al., 2010). Notably, the latter two metabolites are major precursors of acetyl-CoA, indicating that feedforward activation happens to htACC, which stands in opposition to feedback inhibition primarily from 18:1-ACP (Andre et al., 2012).

Moreover, in green tissues, light is a potent environmental stimulator of htACC, so that FA synthesis is coordinated with photosynthesis (Sasaki and Nagano, 2004). Upon illumination, multiple biochemical changes take place in the chloroplast stroma. Those favorable for FA synthesis include the increases in reduced thioredoxin, pH, and the concentrations of Mg^2+^, ATP and NADPH (Sasaki et al., 1997; Hunter and Ohlrogge, 1998). For one thing, reduced thioredoxin, as already mentioned, relieves CT from S-S inhibition, while pH (from 7.0 to 8.0) and Mg^2+^ (from 1 to 3 mM) levels are elevated to the range optimum for the catalytic kinetics of htACC (Sasaki et al., 1997; Hunter and Ohlrogge, 1998; Kozaki et al., 2001); for another, ATP is a substrate for BC and NADPH is the reducing power for FA elongation and desaturation (Salie and Thelen, 2016).

#### 3-Ketoacyl-ACP Synthases

FAS exists in two physically distinct types in nature as well: heteromeric type II and homomeric type I. Plants only have the type II system in plastids and mitochondria, where it is organized from multiple discrete enzymes and a non-catalytic cofactor, i.e., ACP (Yasuno et al., 2004). Of note, ACP is also an essential cofactor for SAD, FAT and plastidial GPAT (Tang et al., 2012). The key catalytic component of FAS is 3-ketoacyl-ACP synthase (KAS) that appends the C2 module via Claisen condensation, the first step in each elongation cycle (Yasuno et al., 2004).

In the biogenesis of 18:0 in plastids, however, three classes of KAS are engaged — KASIII for initial combination between acetyl-CoA and malonyl-ACP, KASI for sequential elongation till 16:0-ACP, and KASII (or FAB1) for additional extension to 18:0-ACP (Ohlrogge and Browse, 1995; Harwood, 1996). Accordingly, KASII activity is a major determinant of the ratio of C18 to C16 FAs in plant cells, as is evident in the *fatty acid biosynthesis 1-1* (*fab1-1*) mutant of *Arabidopsis* that carries a partially defective KASII (Wu et al., 1994; Pidkowich et al., 2007). Moreover, it should be noted that 18:0 can also be synthesized by the mitochondrial FAS utilizing a singular KAS, although its physiological role and metabolic fate remain to be unveiled (Gueguen et al., 2000; Yasuno et al., 2004).

In *Arabidopsis*, each KAS is a single gene product. Apart from chain-length specificity, the three KAS members also differ in sensitivity to inhibitors such as cerulenin and thiolactomycin (Harwood, 1996; Jones et al., 2000). The two antibiotics are most effective against KASI and KASII, respectively. Insensitive to cerulenin as it is, KASIII is subjected to feedback inhibition like ACC, owing to its switch position in the acyl chain assembly. Nevertheless, medium-chain acyl-ACPs (e.g., 10:0-ACP) appear to be stronger effectors in different plant species (Brück et al., 1996; Abbadi et al., 2000), not least *Cuphea*, which deploys a specific KASIV for the manufacture of medium-chain FAs in seeds (Dehesh et al., 1998; Schütt et al., 2002).

#### Fatty Acid Desaturases

By inserting the first double bond into 18:0, SAD is crucial for C18 UFA biogenesis. Accordingly, SAD, as with FATs and acyltransferases, is a major determinant of the homeostasis between unsaturated and saturated FAs. As an archetypal soluble FAD, SAD is normally kinetic in the stroma, keeping 18:0 at a quite low level (Harwood, 1996). In *Arabidopsis*, seven encoding genes have been identified. Now that the endogenous paralogs did not compensate for the *suppressor of SA insensitive 2* (*ssi2*) or *fab2* mutation, SSI2/FAB2 is likely the predominant one engaged in the committed step of C18 unsaturation, which indeed exhibited the highest SAD activity (Kachroo et al., 2007). Impressively, based on the resolved crystal structure of SAD from castor (*Ricinus communis*) (Lindqvist et al., 1996), it is facile to manipulate the functional mode of acyl-ACP desaturases. For example, five amino-acid substitutions sufficiently turned a Δ6 16:0-ACP desaturase into a Δ9 SAD (Cahoon et al., 1997).

The two sets of FADs that catalyze the subsequent unsaturation steps are all integral proteins acting on acyl-lipids. Apart from cellular location and lipid substrate, the two sets also differ in electron donor, with cytochrome b_5_ for the ER FAD2-FAD3 and ferredoxin for the plastidial FAD6-FAD7/8 (Shanklin and Cahoon, 1998). In *Arabidopsis*, each of the five FADs is encoded by a single gene. Of note, in leaves, the ω-6 and ω-3 pair in each set may work as heterodimers to facilitate the metabolic channeling (Lou et al., 2014). Recently, the flippase ALA10 has been shown to interact specifically with FAD2 and may thereby affect the balance between FAD2 and FAD3, leading to the discharging and flipping of 18:2-containing PC (Botella et al., 2016).

C18 UFAs are closely intertwined with stresses as not only general defenders but primary victims; the toxic peroxidation product malondialdehyde (MDA) is widely used as an indicator of oxidative damage (Guo et al., 2012; He et al., 2018). In line with this, broadly speaking, these FADs can be labeled as stress-responsive, transcriptionally and/or post-transcriptionally, albeit the response varies with gene, tissue, species, and/or stress (examples listed in Table 1). Temperature is one of the main environmental factors that influence FAD expression. In most cases, the mRNA and/or protein levels of FADs, if responsive, will change inversely with ambient temperature (Table 1). It appears that protein degradation is a preferred level on which temperature imposes the effect. The amino acid segments accounting for the thermal instability of several FADs have been mapped, including the C-terminal region of FAD8 from *Arabidopsis* (Matsuda et al., 2005), the N-terminal and 241-334 regions of FAD2-1A from soybean (*Glycine max*) (Tang et al., 2005), and the N-terminal region of FAD3 from *Brassica napus* (Khuu et al., 2011). In addition, GmFAD2-1A and -1B have also been observed to undergo Ser185 phosphorylation that may impair their activities (Tang et al., 2005).

**TABLE 1:** Stress responses of plant fatty acid desaturases involved in C18 unsaturated fatty acid synthesis.

**Gene**	**Species**	**Tissue**	**Stress response^a^**	**References**
*SAD*	*Persea americana*	Fruit	Increased mRNA level under LT (4°C), wounding or fungal invasion	Madi et al., 2003
	*Solanum commersonii*	Leaf	Increased mRNA level after cold acclimation	Vega et al., 2004
	*Solanum tuberosum*	Leaf	No notable changes in mRNA level after cold acclimation	Vega et al., 2004
*FAD2*	*Arabidopsis thaliana*	Leaf	No notable changes in mRNA level at LT (6°C)	Okuley et al., 1994
	*Glycine max*	Seed	Decreased protein stability of FAD2-1A at HT (30°C)	Tang et al., 2005
	*Petroselinum crispum*	Leaf	Increased mRNA level under fungal elicitor	Kirsch et al., 1997a
	*Portulaca oleracea*	Leaf	Increased mRNA level of *FAD2-2* under LT (5°C) or wounding	Teixeira et al., 2009
*FAD3*	*Arabidopsis thaliana*	Leaf	Increased mRNA level at LT (4°C)	Kreps et al., 2002
	*Triticum aestivum*	Root tip	Increased protein synthesis with slightly increased mRNA level at LT (10°C)	Horiguchi et al., 1996
	*Brassica napus*	/	Increased protein stability without notable changes in mRNA level at LT (10°C)	O’Quin et al., 2010
	*Vigna radiata*	Hypocotyl	Increased mRNA level under wounding	Yamamoto et al., 1992
*FAD6*	*Arabidopsis thaliana*	Seedling	Increased mRNA level under salt or osmotic stress (300 mM NaCl or mannitol)	Zhang et al., 2009
	*Glycine max*	Leaf	No notable changes in mRNA level with temperature	Heppard et al., 1996
	*Portulaca oleracea*	Leaf	Increased mRNA level under wounding but not LT (5°C)	Teixeira et al., 2009
	*Olea europaea*	Fruit	Decreased mRNA level at HT (35°C); no notable changes under wounding	Hernández et al., 2011
*FAD7*	*Arabidopsis thaliana*	Leaf	No notable changes in mRNA level at HT (30°C)	Gibson et al., 1994
		Leaf, root	Increased mRNA level under wounding	Nishiuchi and Iba, 1998
	*Zea mays*	Leaf	Decreased mRNA level at LT (5°C)	Berberich et al., 1998
		Root	Increased mRNA level under salt (400 mM NaCl)	Berberich et al., 1998
	*Petroselinum crispum*	Leaf	Increased mRNA level under fungal elicitor	Kirsch et al., 1997b
*FAD8*	*Arabidopsis thaliana*	Leaf	Decreased mRNA level at HT (30°C)	Gibson et al., 1994
		Leaf	Decreased protein stability at HT (27°C)	Matsuda et al., 2005
		Leaf, root	Increased mRNA level under wounding	Nishiuchi and Iba, 1998
	*Zea mays*	Leaf	Increased mRNA level at LT (5°C)	Berberich et al., 1998
		Root	Increased mRNA level under salt (400 mM NaCl)	Berberich et al., 1998

*^*a*^LT: low temperature; HT: high temperature.*

#### Glycerol-3-Phosphate Acyltransferases

The biosynthesis of C18 PUFAs being coupled to that of membrane lipids highlights the importance of GPAT. Composing a multigene family *in planta*, GPATs acylate the *sn*-1 or *sn*-2 position of G3P to form lysoPA, which is a common intermediate to membrane glycerolipids, storage TAGs, and extracellular polyesters (e.g., cutin) (Waschburger et al., 2018). In *Arabidopsis*, there are 10 GPAT genes designated *ATS1* (or *ACT1*) and *GPAT1-9*. Their products are distributed to three subcellular compartments (ATS1 to plastids, GPAT1-3 to mitochondria, and GPAT4-9 to the ER) to be involved in different metabolic pathways and biological functions (Chen et al., 2011; Sui et al., 2017; Jayawardhane et al., 2018).

With respect to C18 PUFA generation, ATS1 and GPAT9, the two *sn*-1 regiospecific members, control the entries of the prokaryotic and eukaryotic pathways, respectively (Jayawardhane et al., 2018; Waschburger et al., 2018). Localized in the stroma, ATS1 is the only soluble GPAT member. Interestingly, its orthologs in chilling-resistant plants like spinach (*Spinacia oleracea*) exhibit a pronounced preference for 18:1-ACP (Bertrams and Heinz, 1981). In contrast, those in chilling-sensitive plants like squash (*Cucurbita moschata*) barely discriminate between 18:1-ACP and 16:0-ACP (Frentzen et al., 1987). Bound to the ER membrane, GPAT9 is essential for the production of both membrane and storage lipids. The orthologs have been shown to favor certain substrate as well, for example, 18:1-CoA in *Arabidopsis* (Singer et al., 2016) vs. 16:0-CoA in sunflower (*Helianthus annuus*) (Payá-Milans et al., 2016).

### Transcriptional Regulation and Signaling Pathways

#### Transcriptional Factors

Transcriptional regulation of FA synthetic genes (Figure 2) is more characterized in seeds, the TAG biofactories. Not surprisingly, a subset of enzymes is grouped into a regulon to be coordinately regulated by the same TF, as exemplified by those cited hereafter. The first ‘master regulator’ to be identified is WRINKLED1 (WRI1), whose mutation caused an 80% reduction in seed oil content of *Arabidopsis* (Focks and Benning, 1998). Being a member of the APETALA2/ethylene response factor (AP2/ERF) superfamily of TFs, it functions via binding to the AW boxes harbored in the regulatory regions of the targets (see review Kong and Ma, 2018). To orchestrate carbon flux and energy reserve, WRI1 controls multiple genes involved in glycolysis and FA synthesis. Notably, it activates not only the anabolic genes including *BCCP2*, *ACP3*, *KASI*, and *FAD2* (Ruuska et al., 2002), but the regulatory genes including *BADC1*, *-2*, and *-3* (Liu H. et al., 2019).

WRI1 is situated at a downstream node in an intricate regulatory network that governs seed development. The core regulators comprise ABSCISIC ACID INSENSITIVE 3 (ABI3) and three leafy cotyledon (LEC) group factors, i.e., LEC1, LEC2 and FUSCA3 (FUS3). Of them, LEC1 is a subunit of the heterotrimeric CCAAT-box binding factor (CBF, or NF-Y for nuclear factor Y), while the other three are B3 domain TFs recognizing RY motifs (see reviews Baud and Lepiniec, 2010; Kong and Ma, 2018). Nevertheless, WRI1 is not a direct target of ABI3 in *Arabidopsis* according to the chromatin immunoprecipitation (ChIP) assay (Mönke et al., 2012), and is only critical for specifying the activating role of LEC2 toward the TAG deposit (Baud et al., 2007). Upon overexpression, LEC1 is capable of inducing a larger set of FA synthetic genes, with *CAC2*, *CAC3*, *ACP1*, *KASII*, *SSI2*, *FAD3*, etc. being appended to the list. This more global effect depends largely on FUS3 and partially on WRI1 and ABI3 (Mu et al., 2008). In fact, LEC1 can modulate FA synthesis in a direct manner by targeting *SSI2* (Junker et al., 2012), as can FUS3 by targeting *KASI*, *ACP1*, and *FAD2* (Wang and Perry, 2013).

Accordingly, repressors of these master regulators impose a negative effect on FA biosynthesis. TRANSPARENT TESTA 8 (TT8), for instance, can directly inhibit the transcription of *LEC1*, *LEC2*, and *FUS3* (Chen et al., 2014). This basic helix-loop-helix (bHLH) TF can form a ternary complex with an R2R3-MYB factor TT2 and a WD40 repeat factor TRANSPARENT TESTA GLABRA 1 (TTG1) to monitor carbon partitioning among metabolic pathways that share the same source, including seed oil, mucilage and flavonoid synthesis (Li et al., 2018). Interestingly, there is a TT2-FUS3-TTG1 cascade linked via direct gene suppression (Wang et al., 2014; Chen et al., 2015). Further, TTG1 can indirectly suppress *ABI3*, *LEC2*, *BCCP2*, *CAC2*, *FAD2*, *FAD3* and so on (Chen et al., 2015). Later, MYB89 was identified as a repressor of *WRI1*. It can also inhibit *BCCP1*, *KASI*, *FAD2*, *FAD3*, etc., directly or indirectly (Li et al., 2017).

Members of the zinc finger DOF (DNA binding with one finger) family also participate in FA synthesis regulation (Wang et al., 2007; Deng et al., 2015). In *B. napus*, for example, knockdown of *DOF5.6* resulted in decreased expression of several *KASII* and *SAD* genes. Intriguingly, two *FAD3* genes exhibited different responses. *Bra018348* was down-regulated, whereas *Bra022767* was up-regulated, albeit the outcome was reduced 18:3 level (Deng et al., 2015). In addition, it is worth noting that WAX INDUCER 1/SHINE1 (WIN1/SHN1), another AP2/ERF member known to be important for extracelluar cuticle synthesis in epidermal cells, has its positive effect on intracellular oil yield in seed cells unveiled. In *B. napus*, *BCCP1* and *GPAT9* are among its direct targets (Liu N. et al., 2019).

The investigation of TFs acting in other plant tissues is also ongoing. Based on previous comparative transcriptome analysis, a fruit-specific complex network similar to the seed one has been established in the mesocarp of oil palm (*Elaeis guineensis*). NF-YA3, NF-YC2 and ABI5 directly activate WRI1-1 and a subset of FA synthetic genes. NF-YA3 can also physically interact with NF-YC2, ABI5 and WRI1-1, thereby forming a TF complex to modulate gene expression. WRKY40 then cooperates with WRKY2 in repressing ABI5 and thus oil synthesis (Yeap et al., 2017). Moreover, DREB2C, a third AP2/ERF member classified into subgroup 2 of the dehydration-responsive element-binding TF family, emerged to promote 18:3 production. When transformed into *Arabidopsis* from *Ammopiptanthus mongolicus*, a desert evergreen broadleaf shrub, AmDREB2C could up-regulate *AtFAD3* and *AtFAD8* in siliques and *AtFAD7* in leaves (Yin Y. et al., 2018).

#### Signaling Pathways

The upstream signaling pathways are also emerging (Figure 2). The WRI1 regulon is naturally included in the energy-sensing signalosome centered on SnRK1, a conserved member of the sucrose non-fermenting 1-related protein kinase superfamily (see review Crepin and Rolland, 2019). It appears that SnRK1 is more subject to repression by high energy signals like sugars, leading to metabolic reprogramming from catabolism to anabolism. In the signaling triggered by trehalose 6-phosphate (T6P), an agent of cellular sucrose status, WRI1 will be rescued from the suppression of KIN10, a catalytic α-subunit of SnRK1 (Zhai et al., 2018). KIN10 is enabled to phosphorylate and thus promote the proteolysis of WRI1, once its functional T-loop is phosphorylated by SnRK1-activating kinases (SnAKs, originally GRIKs for geminivirus rep-interacting kinases). However, T6P can directly bind to KIN10, thereby disturbing its affinity for and activation by SnAKs. It is delicate that the photosynthetically fixed carbon *per se* switches on FA synthesis so as to flow into storage. Notably, FUS3 is a substrate of SnRK1 as well; however, in contrast to WRI1, phosphorylation by KIN10 is conducive to its protein stability and TF activity (Tsai and Gazzarrini, 2012; Chan et al., 2016).

The repressor TTG1 is regulated by shaggy-like kinases 11/12 (SK11/12) that belongs to the glycogen synthase kinase-3 (GSK3) family (Li et al., 2018). Of note, SK11 (or ASKα) can be induced by salt stress (Dal Santo et al., 2012). TTG1, together with TT2 and TT8, coordinates carbon partitioning. However, they drive carbon flux into the synthetic pathways of seed coat mucilage and flavonoid pigments, which compete with oil for photosynthates (Li et al., 2018). One of their targets is GLABRA2 (GL2). This homeobox TF is an activator of *MUCILAGE MODIFIED 4* (*MUM4*) that encodes the rhamnose synthase for mucilage biogenesis, and the mutation of either gene resulted in higher seed oil content (Shi et al., 2012). When TTG1 is phosphorylated by SK11/12 at Ser215, its interaction with TT2 is abolished. This compromises recruitment of TTG1 to the *GL2* locus, leading to down-regulation of GL2 and an ultimate reduction of mucilage yield. FA synthesis is then enhanced at least partially due to increased carbon allocation (Li et al., 2018), as occurs upon deficiency of TT4, the chalcone synthase for flavonoid biogenesis. In the *tt4* mutant, *WRI1* and its downstream genes involved in glycolysis and FA synthesis are activated, thus driving the reorientated sugar source into the two pathways (Xuan et al., 2018).

Light is likely to regulate FAD expression in a species-specific manner at least at the transcriptional level. Those stimulated include *FAD7s* of *Arabidopsis* (Nishiuchi et al., 1995) and wheat (*Triticum aestivum*) (Horiguchi et al., 1996), *FAD3* and *FAD8* of soybean (Collados et al., 2006), *FAD2-3* and *-4* of cotton (*Gossypium hirsutum*) (Kargiotidou et al., 2008), and *FAD2-1*, *-2*, and *FAD6* of olive (*Olea europaea*) (Hernández et al., 2011). Potential light-responsive G-box motifs have been identified in common in the predicted promoters of *AtFAD2*, *GhFAD2-3*, and *-4* (Kargiotidou et al., 2008), suggesting a conserved regulatory mechanism. These sites might be recognized by phytochrome interacting factors (PIFs), a subfamily of bHLH TFs, or ELONGATED HYPOCOTYL 5 (HY5), a basic leucine zipper (bZIP) TF. Either of them is regulated by phytochromes, which are photoreceptor proteins that perceive red and far-red light (Zhou et al., 2014; He et al., 2016; Legris et al., 2017). Nevertheless, phytochrome B (PHYB), which is also a thermosensor and can directly bind to G-boxes (see review Delker et al., 2017), exhibited a negative effect on the transcription of *FAD7*, *FAD8*, and *ATS1* in rice (*Oryza sativa*), particularly under chilling stress (Yang et al., 2013). Notably, PHYA can antagonize PHYB in regulating the cold signaling of tomato (*Solanum lycopersicum*) (Wang F. et al., 2016), rendering it a candidate mediator of FAD photoactivation.

FA synthesis is subject to hormonal regulation by abscisic acid (ABA), auxin and JA, to name a few (Shahid et al., 2019). Remarkably, ABA, the central phytohormone that organizes plant development, growth and stress defense, appears to be implicated via multiple accesses: (1) ABI3 and ABI5 that are intrinsic components of ABA signaling. (2) DREB2C that is ABA-inducible and can exert a positive feedback on ABA biosynthesis in *Arabidopsis* (Je et al., 2014). It is worth noting that the *AtDREB2C* promoter was predicted to harbor diverse types of *cis*-acting elements (Sazegari et al., 2015), which are responsive to ABA, JA, SA, heat, cold, and defense, respectively, suggesting that the DREB2C regulon is part of the stress signalosome. (3) SnRK1 that is negatively regulated by type 2C protein phosphatases (PP2Cs) (Rodrigues et al., 2013). Such inhibition can be relieved by ABA upon binding to its receptors, as it does to activate SnRK2 in its own signaling. (4) FUS3 that can augment ABA biosynthesis and receive a protein-stabilizing effect in return (Gazzarrini et al., 2004; Lu et al., 2010). Notably, it offers one more nexus between ABA and SnRK1 pathways. (5) PHYB that appears to counteract ABA (and JA) in various stress responses (He et al., 2018). In tobacco (*Nicotiana tabacum*), for instance, the two can suppress each other’s synthesis to affect salt tolerance (Yang et al., 2018). In addition, LEC1 is a potential integrator of ABA and light signaling pathways, which may act on *PP2CA*, *PIF5* and *HY5*, among others (Junker et al., 2012).

## Conclusion and Perspectives

The enzymatic steps involved in C18 UFA biosynthesis have been well established; however, the eukaryotic pathway has not yet been fully understood, which is complicated by acyl editing and FA/lipid trafficking between the plastid and the ER. Particularly, the consensus concerning how and which eukaryotic species return to the plastid envelope has not been reached. Now, the lysoPC-mediated way for PC translocation is being refuted, turning the involvement of LACS4/9 in ER-to-plastid transport into a puzzle. As one of the basic pathways of the metabolism to produce molecules with multiple biological roles, it is natural that FA synthesis is subject to elaborate regulation involving diverse genetic and biochemical mechanisms, which are far from being resolved. With respect to transcriptional regulation, a number of questions remain to be answered, such as which TFs are master regulators working in vegetative tissues, and how the FAD genes respond to environmental factors, including light and temperature. Noteworthily, comparative transcriptome or gene co-expression network analysis can provide valuable information for deciphering the regulatory networks (Yeap et al., 2017; Shahid et al., 2019; Yang et al., 2019). These high-throughput strategies also lead to the identification of potential microRNAs and long non-coding RNAs that fine-tune the expression of FA synthetic genes at the post-transcriptional level (Wang J. et al., 2016; Yin D.D. et al., 2018).

A better knowledge of the key enzymes and their regulation offers the promise of using physical, biochemical and/or genetic means to manipulate FA composition and increase oil yield, so as to meet the demand for improving the ability of crops to deal with multiple stresses, especially in the context of climatic change and soil salinization (Yuan et al., 2015; He et al., 2018), as well as producing edible oils with higher quality and more oleochemicals for industrial usage. A growing number of successes are being achieved via genetically engineering the key enzymes or TFs. For instance, transgenesis of FAD3 or DREB2C resulted in elevated 18:3 content and ameliorated multistress resistance (Shi et al., 2018; Yin Y. et al., 2018). Interestingly, to achieve high 18:1 level that is good for oil stability and human health, *FAD2* has become a hotspot for targeted disruption by using CRISPR/Cas9, the powerful genome editing tool (Abe et al., 2018; Okuzaki et al., 2018; al Amin et al., 2019; Do et al., 2019). Nevertheless, crop improvement would be facilitated if the culture conditions involving light, temperature, fertilizers, and exogenous phytohormones are optimized for UFA synthesis.

## Author Contributions

All authors listed have made a substantial, direct and intellectual contribution to the work, and approved it for publication.

## Conflict of Interest

The authors declare that the research was conducted in the absence of any commercial or financial relationships that could be construed as a potential conflict of interest.

## References

[B1] Abbadi A. Brummel M. Spener F. (2000). Knockout of the regulatory site of 3-ketoacyl-ACP synthase III enhances short- and medium-chain acyl-ACP synthesis. *Plant J.* 24 1–9. 10.1046/j.1365-313x.2000.00841.x 11029699

[B2] Abe K. Araki E. Suzuki Y. Toki S. Saika H. (2018). Production of high oleic/low linoleic rice by genome editing. *Plant Physiol. Biochem.* 131 58–62. 10.1016/j.plaphy.2018.04.033 29735369

[B3] al Amin N. Ahmad N. Wu N. Pu X. Ma T. Du Y. (2019). CRISPR-Cas9 mediated targeted disruption of FAD2-2 microsomal omega-6 desaturase in soybean (*Glycine max*. L). *BMC Biotechnol.* 19:9 10.1186/s12896-019-0501-2 PMC6350355 30691438

[B4] Andre C. Haslam R. P. Shanklin J. (2012). Feedback regulation of plastidic acetyl-CoA carboxylase by 18:1-acyl carrier protein in *Brassica napus*. *Proc. Natl. Acad. Sci. U.S.A.* 109 10107–10112. 10.1073/pnas.1204604109 22665812 PMC3382543

[B5] Babiychuk E. Vandepoele K. Wissing J. Garcia-Diaz M. De Rycke R. Akbari H. (2011). Plastid gene expression and plant development require a plastidic protein of the mitochondrial transcription termination factor family. *Proc. Natl. Acad. Sci. U.S.A.* 108 6674–6679. 10.1073/pnas.1103442108 21464319 PMC3081001

[B6] Bates P. D. Durrett T. P. Ohlrogge J. B. Pollard M. (2009). Analysis of acyl fluxes through multiple pathways of triacylglycerol synthesis in developing soybean embryos. *Plant Physiol.* 150 55–72. 10.1104/pp.109.137737 19329563 PMC2675710

[B7] Baud S. Lepiniec L. (2010). Physiological and developmental regulation of seed oil production. *Prog. Lipid Res.* 49 235–249. 10.1016/j.plipres.2010.01.001 20102727

[B8] Baud S. Mendoza M. S. To A. Harscoët E. Lepiniec L. Dubreucq B. (2007). WRINKLED1 specifies the regulatory action of LEAFY COTYLEDON2 towards fatty acid metabolism during seed maturation in *Arabidopsis*. *Plant J.* 50 825–838. 10.1111/j.1365-313X.2007.03092.x 17419836

[B9] Berberich T. Harada M. Sugawara K. Kodama H. Iba K. Kusano T. (1998). Two maize genes encoding ω-3 fatty acid desaturase and their differential expression to temperature. *Plant Mol. Biol.* 36 297–306. 10.1023/A:1005993408270 9484441

[B10] Bertrams M. Heinz E. (1981). Positional specificity and fatty acid selectivity of purified sn-glycerol 3-phosphate acyltransferases from chloroplasts. *Plant Physiol.* 68 653–657. 10.1104/pp.68.3.653 16661974 PMC425956

[B11] Bessoule J. J. Testet E. Cassagne C. (1995). Synthesis of phosphatidylcholine in the chloroplast envelope after import of lysophosphatidylcholine from endoplasmic reticulum membranes. *Eur. J. Biochem.* 228 490–497. 10.1111/j.1432-1033.1995.0490n.x 7705366

[B12] Botella C. Jouhet J. Block M. A. (2017). Importance of phosphatidylcholine on the chloroplast surface. *Prog. Lipid Res.* 65 12–23. 10.1016/j.plipres.2016.11.001 27871883

[B13] Botella C. Sautron E. Boudiere L. Michaud M. Dubots E. Yamaryo-Botté Y. (2016). ALA10, a phospholipid flippase, controls FAD2/FAD3 desaturation of phosphatidylcholine in the ER and affects chloroplast lipid composition in *Arabidopsis thaliana*. *Plant Physiol.* 170 1300–1314. 10.1104/pp.15.01557 26620528 PMC4775126

[B14] Brück F. M. Brummel M. Schuch R. Spener F. (1996). In-vitro evidence for feed-back regulation of β-ketoacyl-acyl carrier protein synthase III in medium-chain fatty acid biosynthesis. *Planta* 198 271–278. 10.1007/BF00206253

[B15] Cahoon E. B. Lindqvist Y. Schneider G. Shanklin J. (1997). Redesign of soluble fatty acid desaturases from plants for altered substrate specificity and double bond position. *Proc. Natl. Acad. Sci. U.S.A.* 94 4872–4877. 10.1073/pnas.94.10.4872 9144157 PMC24598

[B16] Chan A. Carianopol C. Tsai A. Y. L. Varatharajah K. Chiu R. S. Gazzarrini S. (2016). SnRK1 phosphorylation of FUSCA3 positively regulates embryogenesis, seed yield, and plant growth at high temperature in *Arabidopsis*. *J. Exp. Biol.* 68 4219–4231. 10.1093/jxb/erx233 PMC5853833 28922765

[B17] Chen M. Xuan L. Wang Z. Zhou L. Li Z. Du X. (2014). TRANSPARENT TESTA8 inhibits seed fatty acid accumulation by targeting several seed development regulators in *Arabidopsis*. *Plant Physiol.* 165 905–916. 10.1104/pp.114.235507 24722549 PMC4044850

[B18] Chen M. Zhang B. Li C. Kulaveerasingam H. Chew F. T. Yu H. (2015). TRANSPARENT TESTA GLABRA1 regulates the accumulation of seed storage reserves in *Arabidopsis*. *Plant Physiol.* 169 391–402. 10.1104/pp.15.00943 26152712 PMC4577430

[B19] Chen X. Snyder C. L. Truksa M. Shah S. Weselake R. J. (2011). *sn*-Glycerol-3-phosphate acyltransferases in plants. *Plant Signal. Behav.* 6 1695–1699. 10.4161/psb.6.11.17777 22057337 PMC3329339

[B20] Collados R. Andreu V. Picorel R. Alfonso M. (2006). A light-sensitive mechanism differently regulates transcription and transcript stability of ω3 fatty-acid desaturases (FAD3, FAD7 and FAD8) in soybean photosynthetic cell suspensions. *FEBS Lett.* 580 4934–4940. 10.1016/j.febslet.2006.07.087 16930600

[B21] Crepin N. Rolland F. (2019). SnRK1 activation, signaling, and networking for energy homeostasis. *Curr. Opin. Plant Biol.* 51 29–36. 10.1016/j.pbi.2019.03.006 31030062

[B22] Dal Santo S. Stampfl H. Krasensky J. Kempa S. Gibon Y. Petutschnig E. (2012). Stress-induced GSK3 regulates the redox stress response by phosphorylating glucose-6-phosphate dehydrogenase in *Arabidopsis*. *Plant Cell* 24 3380–3392. 10.1105/tpc.112.101279 22885737 PMC3462638

[B23] Dehesh K. Edwards P. Fillatti J. Slabaugh M. Byrne J. (1998). KAS IV: a 3-ketoacyl-ACP synthase from *Cuphea* sp. is a medium chain specific condensing enzyme. *Plant J.* 15 383–390. 10.1046/j.1365-313x.1998.00218.x 9750349

[B24] Delker C. van Zanten M. Quint M. (2017). Thermosensing enlightened. *Trends Plant Sci.* 22 185–187. 10.1016/j.tplants.2017.01.007 28173982

[B25] Deng W. Yan F. Zhang X. Tang Y. Yuan Y. (2015). Transcriptional profiling of canola developing embryo and identification of the important roles of *BnDof5.6* in embryo development and fatty acids synthesis. *Plant Cell Physiol.* 56 1624–1640. 10.1093/pcp/pcv074 26092973

[B26] Do P. T. Nguyen C. X. Bui H. T. Tran L. T. N. Stacey G. Gillman J. D. (2019). Demonstration of highly efficient dual gRNA CRISPR/Cas9 editing of the homeologous *GmFAD2-1A* and *GmFAD2-1B* genes to yield a high oleic, low linoleic and α-linolenic acid phenotype in soybean. *BMC Plant Biol.* 19:311 10.1186/s12870-019-1906-8 PMC6632005 31307375

[B27] Feria Bourrellier A. B. Valot B. Guillot A. Ambard-Bretteville F. Vidal J. Hodges M. (2010). Chloroplast acetyl-CoA carboxylase activity is 2-oxoglutarate-regulated by interaction of PII with the biotin carboxyl carrier subunit. *Proc. Natl. Acad. Sci. U.S.A.* 107 502–507. 10.1073/pnas.0910097107 20018655 PMC2806706

[B28] Focks N. Benning C. (1998). *wrinkled1*: a novel, low-seed-oil mutant of *Arabidopsis* with a deficiency in the seed-specific regulation of carbohydrate metabolism. *Plant Physiol.* 118 91–101. 10.1104/pp.118.1.91 9733529 PMC34877

[B29] Frentzen M. Nishida I. Murata N. (1987). Properties of the plastidial acyl-(acyl-carrier-protein): glycerol-3-phosphate acyltransferase from the chilling-sensitive plant squash (*Cucurbita moschata*). *Plant Cell Physiol.* 28 1195–1201. 10.1093/oxfordjournals.pcp.a077406

[B30] Gazzarrini S. Tsuchiya Y. Lumba S. Okamoto M. McCourt P. (2004). The transcription factor FUSCA3 controls developmental timing in *Arabidopsis* through the hormones gibberellin and abscisic acid. *Dev. Cell* 7 373–385. 10.1016/j.devcel.2004.06.017 15363412

[B31] Gerhardt E. C. Rodrigues T. E. Müller-Santos M. Pedrosa F. O. Souza E. M. Forchhammer K. (2015). The bacterial signal transduction protein GlnB regulates the committed step in fatty acid biosynthesis by acting as a dissociable regulatory subunit of acetyl-CoA carboxylase. *Mol. Microbiol.* 95 1025–1035. 10.1111/mmi.12912 25557370

[B32] Gibson S. Arondel V. Iba K. Somerville C. (1994). Cloning of a temperature-regulated gene encoding a chloroplast [omega]-3 desaturase from *Arabidopsis thaliana*. *Plant Physiol.* 106 1615–1621. 10.1104/pp.106.4.1615 7846164 PMC159705

[B33] Gueguen V. Macherel D. Jaquinod M. Douce R. Bourguignon J. (2000). Fatty acid and lipoic acid biosynthesis in higher plant mitochondria. *J. Biol. Chem.* 275 5016–5025. 10.1074/jbc.275.7.5016 10671542

[B34] Guo Y. H. Jia W. J. Song J. Wang D. A. Chen M. Wang B. S. (2012). *Thellungilla halophila* is more adaptive to salinity than *Arabidopsis thaliana* at stages of seed germination and seedling establishment. *Acta Physiol. Plant.* 34 1287–1294. 10.1007/s11738-012-0925-y

[B35] Harwood J. L. (1988). Fatty acid metabolism. *Annu. Rev. Plant Physiol. Plant Mol. Biol.* 39 101–138. 10.1146/annurev.pp.39.060188.000533

[B36] Harwood J. L. (1996). Recent advances in the biosynthesis of plant fatty acids. *Biochim. Biophys. Acta* 1301 7–56. 10.1016/0005-2760(95)00242-1 8652653

[B37] He M. He C. Q. Ding N. Z. (2018). Abiotic stresses: general defenses of land plants and chances for engineering multistress tolerance. *Front. Plant Sci.* 9:1771 10.3389/fpls.2018.01771 PMC6292871 30581446

[B38] He Y. A. Li Y. P. Cui L. X. Xie L. X. Zheng C. K. Zhou G. H. (2016). Phytochrome B negatively affects cold tolerance by regulating *OsDREB1* gene expression through Phytochrome interacting factor-like protein OsPIL16 in rice. *Front. Plant Sci.* 7:1963 10.3389/fpls.2016.01963 PMC5183628 28083003

[B39] Heppard E. P. Kinney A. J. Stecca K. L. Miao G. H. (1996). Developmental and growth temperature regulation of two different microsomal ω-6 desaturase genes in soybean. *Plant Physiol.* 110 311–319. 10.1104/pp.110.1.311 8587990 PMC157722

[B40] Hernández M. L. Padilla M. N. Sicardo M. D. Mancha M. Martínez-Rivas J. M. (2011). Effect of different environmental stresses on the expression of oleate desaturase genes and fatty acid composition in olive fruit. *Phytochemistry* 72 178–187. 10.1016/j.phytochem.2010.11.026 21194717

[B41] Hölzl G. Dörmann P. (2019). Chloroplast lipids and their biosynthesis. *Annu. Rev. Plant Biol.* 70 51–81. 10.1146/annurev-arplant-050718-100202 30786236

[B42] Horiguchi G. Iwakawa H. Kodama H. Kawakami N. Nishimura M. Iba K. (1996). Expression of a gene for plastid ω-3 fatty acid desaturase and changes in lipid and fatty acid compositions in light- and dark-grown wheat leaves. *Physiol. Plant.* 96 275–283. 10.1111/j.1399-3054.1996.tb00214.x

[B43] Hsiao A. S. Haslam R. P. Michaelson L. V. Liao P. Chen Q. F. Sooriyaarachchi S. (2014). *Arabidopsis* cytosolic acyl-CoA-binding proteins ACBP4, ACBP5 and ACBP6 have overlapping but distinct roles in seed development. *Biosci. Rep.* 34:e00165 10.1042/BSR20140139 PMC4274664 25423293

[B44] Hunter S. C. Ohlrogge J. B. (1998). Regulation of spinach chloroplast acetyl-CoA carboxylase. *Arch. Biochem. Biophys.* 359 170–178. 10.1006/abbi.1998.0900 9808758

[B45] Hurlock A. K. Wang K. Takeuchi T. Horn P. J. (2018). *In vivo* lipid ‘tag and track’ approach shows acyl editing of plastid lipids and chloroplast import of phosphatidylglycerol precursors in *Arabidopsis thaliana*. *Plant J.* 95 1129–1139. 10.1111/tpj.13999 29920824

[B46] Jacques F. Rippa S. Perrin Y. (2018). Physiology of L-carnitine in plants in light of the knowledge in animals and microorganisms. *Plant Sci.* 274 432–440. 10.1016/j.plantsci.2018.06.020 30080631

[B47] Jayawardhane K. N. Singer S. D. Weselake R. J. Chen G. (2018). Plant *sn*-glycerol-3-phosphate acyltransferases: biocatalysts involved in the biosynthesis of intracellular and extracellular lipids. *Lipids* 53 469–480. 10.1002/lipd.12049 29989678

[B48] Je J. Chen H. Song C. Lim C. O. (2014). *Arabidopsis* DREB2C modulates ABA biosynthesis during germination. *Biochem. Biophys. Res. Commun.* 452 91–98. 10.1016/j.bbrc.2014.08.052 25150152

[B49] Jessen D. Roth C. Wiermer M. Fulda M. (2015). Two activities of long-chain acyl-coenzyme A synthetase are involved in lipid trafficking between the endoplasmic reticulum and the plastid in *Arabidopsis*. *Plant Physiol.* 167 351–366. 10.1104/pp.114.250365 25540329 PMC4326746

[B50] Jones A. L. Herbert D. Rutter A. J. Dancer J. E. Harwood J. L. (2000). Novel inhibitors of the condensing enzymes of the type II fatty acid synthase of pea (*Pisum sativum*). *Biochem. J.* 347 205–209. 10.1042/bj3470205 10727420 PMC1220949

[B51] Junker A. Mönke G. Rutten T. Keilwagen J. Seifert M. Thi T. M. (2012). Elongation-related functions of LEAFY COTYLEDON1 during the development of *Arabidopsis thaliana*. *Plant J.* 71 427–442. 10.1111/j.1365-313X.2012.04999.x 22429691

[B52] Kachroo A. Shanklin J. Whittle E. Lapchyk L. Hildebrand D. Kachroo P. (2007). The *Arabidopsis* stearoyl-acyl carrier protein-desaturase family and the contribution of leaf isoforms to oleic acid synthesis. *Plant Mol. Biol.* 63 257–271. 10.1007/s11103-006-9086-y 17072561

[B53] Kachroo P. Shanklin J. Shah J. Whittle E. J. Klessig D. F. (2001). A fatty acid desaturase modulates the activation of defense signaling pathways in plants. *Proc. Natl. Acad. Sci. U.S.A.* 98 9448–9453. 10.1073/pnas.151258398 11481500 PMC55441

[B54] Kargiotidou A. Deli D. Galanopoulou D. Tsaftaris A. Farmaki T. (2008). Low temperature and light regulate *delta 12* fatty acid desaturases (FAD2) at a transcriptional level in cotton (*Gossypium hirsutum*). *J. Exp. Bot.* 59 2043–2056. 10.1093/jxb/ern065 18453533 PMC2413273

[B55] Karki N. Johnson B. S. Bates P. D. (2019). Metabolically distinct pools of phosphatidylcholine are involved in trafficking of fatty acids out of and into the chloroplast for membrane production. *Plant Cell* 31 2768–2788. 10.1105/tpc.19.00121 31511316 PMC6881139

[B56] Keereetaweep J. Liu H. Zhai Z. Shanklin J. (2018). Biotin attachment domain-containing proteins irreversibly inhibit acetyl CoA carboxylase. *Plant Physiol.* 177 208–215. 10.1104/pp.18.00216 29626162 PMC5933113

[B57] Khuu N. Gidda S. Shockey J. M. Dyer J. M. Mullen R. T. (2011). The N termini of Brassica and tung omega-3 fatty acid desaturases mediate proteasome-dependent protein degradation in plant cells. *Plant Signal. Behav.* 6 422–425. 10.4161/psb.6.3.14522 21350343 PMC3142428

[B58] Kirsch C. Hahlbrock K. Somssich I. E. (1997a). Rapid and transient induction of a parsley microsomal Δ12 fatty acid desaturase mRNA by fungal elicitor. *Plant Physiol.* 115 283–289. 10.1104/pp.115.1.283 9306702 PMC158484

[B59] Kirsch C. Takamiya-Wik M. Reinold S. Hahlbrock K. Somssich I. E. (1997b). Rapid, transient, and highly localized induction of plastidial ω-3 fatty acid desaturase mRNA at fungal infection sites in *Petroselinum crispum*. *Proc. Natl. Acad. Sci. U.S.A.* 94 2079–2084. 10.1073/pnas.94.5.2079 9050908 PMC20046

[B60] Kong Q. Ma W. (2018). WRINKLED1 transcription factor: how much do we know about its regulatory mechanism? *Plant Sci.* 272 153–156. 10.1016/j.plantsci.2018.04.013 29807586

[B61] Konishi T. Sasaki Y. (1994). Compartmentalization of two forms of acetyl-CoA carboxylase in plants and the origin of their tolerance toward herbicides. *Proc. Natl. Acad. Sci. U.S.A.* 91 3598–3601. 10.1073/pnas.91.9.3598 7909603 PMC43627

[B62] Konishi T. Shinohara K. Yamada K. Sasaki Y. (1996). Acetyl-CoA carboxylase in higher plants: most plants other than Gramineae have both the prokaryotic and the eukaryotic forms of this enzyme. *Plant Cell Physiol.* 37 117–122. 10.1093/oxfordjournals.pcp.a028920 8665091

[B63] Kozaki A. Mayumi K. Sasaki Y. (2001). Thiol-disulfide exchange between nuclear-encoded and chloroplast-encoded subunits of pea acetyl-CoA carboxylase. *J. Biol. Chem.* 276 39919–39925. 10.1074/jbc.M103525200 11546765

[B64] Kreps J. A. Wu Y. Chang H. S. Zhu T. Wang X. Harper J. F. (2002). Transcriptome changes for *Arabidopsis* in response to salt, osmotic, and cold stress. *Plant Physiol.* 130 2129–2141. 10.1104/pp.008532 12481097 PMC166725

[B65] LaBrant E. Barnes A. C. Roston R. L. (2018). Lipid transport required to make lipids of photosynthetic membranes. *Photosynth. Res.* 138 345–360. 10.1007/s11120-018-0545-5 29961189

[B66] Lager I. Glab B. Eriksson L. Chen G. Banas A. Stymne S. (2015). Novel reactions in acyl editing of phosphatidylcholine by lysophosphatidylcholine transacylase (LPCT) and acyl-CoA:glycerophosphocholine acyltransferase (GPCAT) activities in microsomal preparations of plant tissues. *Planta* 241 347–358. 10.1007/s00425-014-2184-1 25298156 PMC4302238

[B67] Legris M. Nieto C. Sellaro R. Prat S. Casal J. J. (2017). Perception and signalling of light and temperature cues in plants. *Plant J.* 90 683–697. 10.1111/tpj.13467 28008680

[B68] Li C. Zhang B. Chen B. Ji L. Yu H. (2018). Site-specific phosphorylation of TRANSPARENT TESTA GLABRA1 mediates carbon partitioning in *Arabidopsis* seeds. *Nat. Commun.* 9 571 10.1038/s41467-018-03013-5 PMC5805785 29422671

[B69] Li D. Jin C. Duan S. Zhu Y. Qi S. Liu K. (2017). MYB89 transcription factor represses seed oil accumulation. *Plant Physiol.* 173 1211–1225. 10.1104/pp.16.01634 27932421 PMC5291041

[B70] Li N. Gügel I. L. Giavalisco P. Zeisler V. Schreiber L. Soll J. (2015). FAX1, a novel membrane protein mediating plastid fatty acid export. *PLoS Biol.* 13:e1002053 10.1371/journal.pbio.1002053 PMC4344464 25646734

[B71] Li N. Meng H. Li S. Zhang Z. Zhao X. Wang S. (2020). Two novel plastid fatty acid exporters contribute to seed oil accumulation in *Arabidopsis*. *Plant Physiol.* 182 1910–1919. 10.1104/pp.19.01344 32019874 PMC7140923

[B72] Li N. Xu C. Li-Beisson Y. Philippar K. (2016). Fatty acid and lipid transport in plant cells. *Trends Plant Sci.* 21 145–158. 10.1016/j.tplants.2015.10.011 26616197

[B73] Lim G. H. Singhal R. Kachroo A. Kachroo P. (2017). Fatty acid- and lipid-mediated signaling in plant defense. *Annu. Rev. Phytopathol.* 55 505–536. 10.1146/annurev-phyto-080516-035406 28777926

[B74] Lindqvist Y. Huang W. Schneider G. Shanklin J. (1996). Crystal structure of delta9 stearoyl-acyl carrier protein desaturase from castor seed and its relationship to other di-iron proteins. *EMBO J.* 15 4081–4092. 10.1002/j.1460-2075.1996.tb00783.x 8861937 PMC452130

[B75] Liu H. Zhai Z. Kuczynski K. Keereetaweep J. Schwender J. Shanklin J. (2019). WRINKLED1 regulates BIOTIN ATTACHMENT DOMAIN-CONTAINING proteins that inhibit fatty acid synthesis. *Plant Physiol.* 181 55–62. 10.1104/pp.19.00587 31209126 PMC6716254

[B76] Liu N. Chen J. Wang T. Li Q. Cui P. Jia C. (2019). Overexpression of WAX INDUCER1/SHINE1 gene enhances wax accumulation under osmotic stress and oil synthesis in *Brassica napus*. *Int. J. Mol. Sci.* 20:4435 10.3390/ijms20184435 PMC6771042 31505838

[B77] Lou Y. Schwender J. Shanklin J. (2014). FAD2 and FAD3 desaturases form heterodimers that facilitate metabolic channeling *in vivo*. *J. Biol. Chem.* 289 17996–18007. 10.1074/jbc.M114.572883 24811169 PMC4140268

[B78] Lu B. Benning C. (2009). A 25-amino acid sequence of the *Arabidopsis* TGD2 protein is sufficient for specific binding of phosphatidic acid. *J. Biol. Chem.* 284 17420–17427. 10.1074/jbc.M109.016014 19416982 PMC2719382

[B79] Lu Q. S. dela Paz J. Pathmanathan A. Chiu R. S. Tsai A. Y. L. Gazzarrini S. (2010). The C-terminal domain of FUSCA3 negatively regulates mRNA and protein levels, and mediates sensitivity to the hormones abscisic acid and gibberellic acid in *Arabidopsis*. *Plant J.* 64 100–113. 10.1111/j.1365-313X.2010.04307.x 20663088

[B80] Madi L. Wang X. Kobiler I. Lichter A. Prusky D. (2003). Stress on avocado fruits regulates Δ9-stearoyl ACP desaturase expression, fatty acid composition, antifungal diene level and resistance to *Colletotrichum gloeosporioides* attack. *Physiol. Mol. Plant Pathol.* 62 277–283. 10.1016/S0885-5765(03)00076-6

[B81] Madoka Y. Tomizawa K. Mizoi J. Nishida I. Nagano Y. Sasaki Y. (2002). Chloroplast transformation with modified *accD* operon increases acetyl-CoA carboxylase and causes extension of leaf longevity and increase in seed yield in tobacco. *Plant Cell Physiol.* 43 1518–1525. 10.1093/pcp/pcf172 12514249

[B82] Matsuda O. Sakamoto H. Hashimoto T. Iba K. (2005). A temperature-sensitive mechanism that regulates post-translational stability of a plastidial omega-3 fatty acid desaturase (FAD8) in *Arabidopsis* leaf tissues. *J. Biol. Chem.* 280 3597–3604. 10.1074/jbc.M407226200 15545277

[B83] Mongrand S. Cassagne C. Bessoule J. J. (2000). Import of lyso-phosphatidylcholine into chloroplasts likely at the origin of eukaryotic plastidial lipids. *Plant Physiol.* 122 845–852. 10.1104/pp.122.3.845 10712548 PMC58920

[B84] Mönke G. Seifert M. Keilwagen J. Mohr M. Grosse I. Hähnel U. (2012). Toward the identification and regulation of the *Arabidopsis thaliana* ABI3 regulon. *Nucleic Acids Res.* 40 8240–8254. 10.1093/nar/gks594 22730287 PMC3458547

[B85] Mu J. Tan H. Zheng Q. Fu F. Liang Y. Zhang J. (2008). LEAFY COTYLEDON1 is a key regulator of fatty acid biosynthesis in *Arabidopsis*. *Plant Physiol.* 148 1042–1054. 10.1104/pp.108.126342 18689444 PMC2556827

[B86] Nishiuchi T. Iba K. (1998). Roles of plastid ω-3 fatty acid desaturases in defense response of higher plants. *J. Plant Res.* 111 481–486. 10.1007/BF02507782

[B87] Nishiuchi T. Nakamura T. Abe T. Kodama H. Nishimura M. Iba K. (1995). Tissue-specific and light-responsive regulation of the promoter region of the *Arabidopsis thaliana* chloroplast ω-3 fatty acid desaturase gene (*FAD7*). *Plant Mol. Biol.* 29 599–609. 10.1007/BF00020987 8534855

[B88] Ohlrogge J. Browse J. (1995). Lipid biosynthesis. *Plant Cell* 7 957–970. 10.1105/tpc.7.7.957 7640528 PMC160893

[B89] Okuley J. Lightner J. Feldmann K. Yadav N. Lark E. Browse J. (1994). *Arabidopsis FAD2* gene encodes the enzyme that is essential for polyunsaturated lipid synthesis. *Plant Cell* 6 147–158. 10.1105/tpc.6.1.147 7907506 PMC160423

[B90] Okuzaki A. Ogawa T. Koizuka C. Kaneko K. Inaba M. Imamura J. (2018). CRISPR/Cas9-mediated genome editing of the fatty acid desaturase 2 gene in *Brassica Napus*. *Plant Physiol. Biochem.* 131 63–69. 10.1016/j.plaphy.2018.04.025 29753601

[B91] O’Quin J. B. Bourassa L. Zhang D. Shockey J. M. Gidda S. K. Fosnot S. (2010). Temperature-sensitive, posttranslational regulation of plant omega-3 fatty acid destaturases is mediated by the ER-associated degradation pathway. *J. Biol. Chem.* 285 21781–21796. 10.1074/jbc.M110.135236 20452984 PMC2898375

[B92] Payá-Milans M. Aznar-Moreno J. A. Balbuena T. S. Haslam R. P. Gidda S. K. Pérez-Hormaeche J. (2016). Sunflower HaGPAT9-1 is the predominant GPAT during seed development. *Plant Sci.* 252 42–52. 10.1016/j.plantsci.2016.07.002 27717477

[B93] Pidkowich M. S. Nguyen H. T. Heilmann I. Ischebeck T. Shanklin J. (2007). Modulating seed beta-ketoacyl-acyl carrier protein synthase II level converts the composition of a temperate seed oil to that of a palm-like tropical oil. *Proc. Natl. Acad. Sci. U.S.A.* 104 4742–4747. 10.1073/pnas.0611141104 17360594 PMC1838670

[B94] Rodrigues A. Adamo M. Crozet P. Margalha L. Confraria A. Martinho C. (2013). ABI1 and PP2CA phosphatases are negative regulators of Snf1-related protein kinase1 signaling in *Arabidopsis*. *Plant Cell* 25 3871–3884. 10.1105/tpc.113.114066 24179127 PMC3877788

[B95] Rousseau-Gueutin M. Huang X. Higginson E. Ayliffe M. Day A. Timmis J. N. (2013). Potential functional replacement of the plastidic acetyl-CoA carboxylase subunit (*accD*) gene by recent transfers to the nucleus in some angiosperm lineages. *Plant Physiol.* 161 1918–1929. 10.1104/pp.113.214528 23435694 PMC3613465

[B96] Ruuska S. A. Girke T. Benning C. Ohlrogge J. B. (2002). Contrapuntal networks of gene expression during *Arabidopsis* seed filling. *Plant Cell* 14 1191–1206. 10.1105/tpc.000877 12084821 PMC150774

[B97] Salie M. J. Thelen J. J. (2016). Regulation and structure of the heteromeric acetyl-CoA carboxylase. *Biochim. Biophys. Acta* 1861 1207–1213. 10.1016/j.bbalip.2016.04.004 27091637

[B98] Salie M. J. Zhang N. Lancikova V. Xu D. Thelen J. J. (2016). A family of negative regulators targets the committed step of *de novo* fatty acid biosynthesis. *Plant Cell* 28 2312–2325. 10.1105/tpc.16.00317 27559025 PMC5059801

[B99] Sasaki Y. Kozaki A. Hatano M. (1997). Link between light and fatty acid synthesis: thioredoxin-linked reductive activation of plastidic acetyl-CoA carboxylase. *Proc. Natl. Acad. Sci. U.S.A.* 94 11096–11101. 10.1073/pnas.94.20.11096 9380765 PMC23628

[B100] Sasaki Y. Kozaki A. Ohmori A. Iguchi H. Nagano Y. (2001). Chloroplast RNA editing required for functional acetyl-CoA carboxylase in plants. *J. Biol. Chem.* 276 3937–3940. 10.1074/jbc.M008166200 11078738

[B101] Sasaki Y. Nagano Y. (2004). Plant acetyl-CoA carboxylase: structure, biosynthesis, regulation, and gene manipulation for plant breeding. *Biosci. Biotechnol. Biochem.* 68 1175–1184. 10.1271/bbb.68.1175 15215578

[B102] Savage L. J. Ohlrogge J. B. (1999). Phosphorylation of pea chloroplast acetyl-CoA carboxylase. *Plant J.* 18 521–527. 10.1046/j.1365-313X.1999.00478.x 10417702

[B103] Sazegari S. Niazi A. Ahmadi F. S. (2015). A study on the regulatory network with promoter analysis for *Arabidopsis DREB*-genes. *Bioinformation* 11 101–106. 10.6026/97320630011101 25848171 PMC4369686

[B104] Schütt B. S. Abbadi A. Loddenkötter B. Brummel M. Spener F. (2002). Beta-ketoacyl-acyl carrier protein synthase IV: a key enzyme for regulation of medium-chain fatty acid synthesis in *Cuphea lanceolata* seeds. *Planta* 215 847–854. 10.1007/s00425-002-0803-8 12244451

[B105] Shahid M. Cai G. Zu F. Zhao Q. Qasim M. U. Hong Y. (2019). Comparative transcriptome analysis of developing seeds and silique wall reveals dynamic transcription networks for effective oil production in *Brassica napus* L. *Int. J. Mol. Sci.* 20:1982 10.3390/ijms20081982 PMC6515390 31018533

[B106] Shanklin J. Cahoon E. B. (1998). Desaturation and related modifications of fatty acids. *Annu. Rev. Plant Physiol. Plant Mol. Biol.* 49 611–641. 10.1146/annurev.arplant.49.1.611 15012248

[B107] Shi L. Katavic V. Yu Y. Kunst L. Haughn G. (2012). *Arabidopsis glabra2* mutant seeds deficient in mucilage biosynthesis produce more oil. *Plant J.* 69 37–46. 10.1111/j.1365-313X.2011.04768.x 21883555

[B108] Shi Y. Yue X. An L. (2018). Integrated regulation triggered by a cryophyte ω-3 desaturase gene confers multiple-stress tolerance in tobacco. *J. Exp. Bot.* 69 2131–2148. 10.1093/jxb/ery050 29432580 PMC6019038

[B109] Shintani D. Roesler K. Shorrosh B. Savage L. Ohlrogge J. (1997). Antisense expression and overexpression of biotin carboxylase in tobacco leaves. *Plant Physiol.* 114 881–886. 10.1104/pp.114.3.881 9232874 PMC158375

[B110] Shivaiah K. K. Ding G. Upton B. Nikolau B. J. (2020). Non-catalytic subunits facilitate quaternary organization of plastidic acetyl-CoA carboxylase. *Plant Physiol.* 182 756–775. 10.1104/pp.19.01246 31792149 PMC6997691

[B111] Singer S. D. Chen G. Mietkiewska E. Tomasi P. Jayawardhane K. Dyer J. M. (2016). *Arabidopsis* GPAT9 contributes to synthesis of intracellular glycerolipids but not surface lipids. *J. Exp. Biol.* 67 4627–4638. 10.1093/jxb/erw242 PMC4973736 27325892

[B112] Sudianto E. Chaw S. M. (2019). Two independent plastid *accD* transfers to the nuclear genome of *Gnetum* and other insights on acetyl-CoA carboxylase evolution in gymnosperms. *Genome Biol. Evol.* 11 1691–1705. 10.1093/gbe/evz059 30924880 PMC6595918

[B113] Sui N. Tian S. S. Wang W. Q. Wang M. J. Fan H. (2017). Overexpression of glycerol-3-phosphate acyltransferase from *Suaeda salsa* improves salt tolerance in *Arabidopsis*. *Front. Plant Sci.* 8:1337 10.3389/fpls.2017.01337 PMC5539759 28824673

[B114] Tang G. Q. Novitzky W. P. Carol Griffin H. Huber S. C. Dewey R. E. (2005). Oleate desaturase enzymes of soybean: evidence of regulation through differential stability and phosphorylation. *Plant J.* 44 433–446. 10.1111/j.1365-313X.2005.02535.x 16236153

[B115] Tang G. Y. Wei L. Q. Liu Z. J. Bi Y. P. Shan L. (2012). Ectopic expression of peanut acyl carrier protein in tobacco alters fatty acid composition in the leaf and resistance to cold stress. *Biol. Plant.* 56 493–501. 10.1007/s10535-012-0057-7

[B116] Teixeira M. C. Coelho N. Olsson M. E. Brodelius P. E. Carvalho I. S. Brodelius M. (2009). Molecular cloning and expression analysis of three omega-6 desaturase genes from purslane (*Portulaca oleracea* L.). *Biotechnol. Lett.* 31 1089–1101. 10.1007/s10529-009-9956-x 19277477

[B117] Thelen J. J. Ohlrogge J. B. (2002). Both antisense and sense expression of biotin carboxyl carrier protein isoform 2 inactivates the plastid acetyl-coenzyme A carboxylase in *Arabidopsis thaliana*. *Plant J.* 32 419–431. 10.1046/j.1365-313X.2002.01435.x 12445115

[B118] Tian Y. Lv X. Xie G. Wang L. Dai T. Qin X. (2019). FAX2 mediates fatty acid export from plastids in developing *Arabidopsis* seeds. *Plant Cell Physiol.* 60 2231–2242. 10.1093/pcp/pcz117 31198959

[B119] Tsai A. Y. L. Gazzarrini S. (2012). AKIN10 and FUSCA3 interact to control lateral organ development and phase transitions in *Arabidopsis*. *Plant J.* 69 809–821. 10.1111/j.1365-313X.2011.04832.x 22026387

[B120] Vega S. E. del Rio A. H. Bamberg J. B. Palta J. P. (2004). Evidence for the up-regulation of stearoyl-ACP (Δ9) desaturase gene expression during cold acclimation. *Am. J. Pot. Res.* 81 125–135. 10.1007/BF02853610

[B121] Wang F. Guo Z. Li H. Wang M. Onac E. Zhou J. (2016). Phytochrome A and B function antagonistically to regulate cold tolerance via abscisic acid-dependent jasmonate signaling. *Plant Physiol.* 170 459–471. 10.1104/pp.15.01171 26527654 PMC4704577

[B122] Wang J. Jian H. Wang T. Wei L. Li J. Li C. (2016). Identification of microRNAs actively involved in fatty acid biosynthesis in developing *Brassica napus* seeds using high-throughput sequencing. *Front. Plant Sci.* 7:1570 10.3389/fpls.2016.01570 PMC5075540 27822220

[B123] Wang F. Perry S. E. (2013). Identification of direct targets of FUSCA3, a key regulator of *Arabidopsis* seed development. *Plant Physiol.* 161 1251–1264. 10.1104/pp.112.212282 23314941 PMC3585594

[B124] Wang H. W. Zhang B. Hao Y. J. Huang J. Tian A. G. Liao Y. (2007). The soybean Dof-type transcription factor genes, *GmDof4* and *GmDof11*, enhance lipid content in the seeds of transgenic *Arabidopsis* plants. *Plant J.* 52 716–729. 10.1111/j.1365-313X.2007.03268.x 17877700

[B125] Wang L. Shen W. Kazachkov M. Chen G. Chen Q. Carlsson A. S. (2012). Metabolic interactions between the Lands cycle and the Kennedy pathway of glycerolipid synthesis in *Arabidopsis* developing seeds. *Plant Cell* 24 4652–4669. 10.1105/tpc.112.104604 23150634 PMC3531858

[B126] Wang Z. Xu C. Benning C. (2012). TGD4 involved in endoplasmic reticulum-to-chloroplast lipid trafficking is a phosphatidic acid binding protein. *Plant J.* 70 614–623. 10.1111/j.1365-313X.2012.04900.x 22269056

[B127] Wang Z. Chen M. Chen T. Xuan L. Li Z. Du X. (2014). TRANSPARENT TESTA2 regulates embryonic fatty acid biosynthesis by targeting FUSCA3 during the early developmental stage of *Arabidopsis* seeds. *Plant J.* 77 757–769. 10.1111/tpj.12426 24397827

[B128] Waschburger E. Kulcheski F. R. Veto N. M. Margis R. Margis-Pinheiro M. Turchetto-Zolet A. C. (2018). Genome-wide analysis of the Glycerol-3-Phosphate Acyltransferase (GPAT) gene family reveals the evolution and diversification of plant GPATs. *Genet. Mol. Biol.* 41 355–370. 10.1590/1678-4685-GMB-2017-0076 29583156 PMC5913721

[B129] Wu J. James D. W. J. Dooner H. K. Browse J. (1994). A mutant of *Arabidopsis* deficient in the elongation of palmitic acid. *Plant Physiol.* 106 143–150. 10.1104/pp.106.1.143 12232312 PMC159509

[B130] Xuan L. Zhang C. Yan T. Wu D. Hussain N. Li Z. (2018). TRANSPARENT TESTA 4-mediated flavonoids negatively affect embryonic fatty acid biosynthesis in *Arabidopsis*. *Plant Cell Environ.* 41 2773–2790. 10.1111/pce.13402 29981254

[B131] Yamamoto K. T. Mori H. Imaseki H. (1992). Novel mRNA sequences induced by indole-3-acetic acid in sections of elongating hypocotyls of mung bean (*Vigna radiata*). *Plant Cell Physiol.* 33 13–20. 10.1093/oxfordjournals.pcp.a078214

[B132] Yang J. C. Li M. Xie X. Z. Han G. L. Sui N. Wang B. S. (2013). Deficiency of phytochrome B alleviates chilling-induced photoinhibition in rice. *Am. J. Bot.* 100 1860–1870. 10.3732/ajb.1200574 24018854

[B133] Yang S. Miao L. He J. Zhang K. Li Y. Gai J. (2019). Dynamic transcriptome changes related to oil accumulation in developing soybean seeds. *Int. J. Mol. Sci.* 20:2202 10.3390/ijms20092202 PMC6539092 31060266

[B134] Yang T. Lv R. Li J. Lin H. Xi D. (2018). Phytochrome A and B negatively regulate salt stress tolerance of *Nicotiana tobacum* via abscisic acid-jasmonic acid synergistic cross talk. *Plant Cell Physiol.* 59 2381–2393. 10.1093/pcp/pcy164 30124925

[B135] Yasuno R. von Wettstein-Knowles P. Wada H. (2004). Identification and molecular characterization of the β-ketoacyl-[acyl carrier protein] synthase component of the *Arabidopsis* mitochondrial fatty acid synthase. *J. Biol. Chem.* 279 8242–8251. 10.1074/jbc.M308894200 14660674

[B136] Yeap W. C. Lee F. C. Shabari Shan D. K. Musa H. Appleton D. R. Kulaveerasingam H. (2017). WRI1-1, ABI5, NF-YA3 and NF-YC2 increase oil biosynthesis in coordination with hormonal signaling during fruit development in oil palm. *Plant J.* 91 97–113. 10.1111/tpj.13549 28370622

[B137] Yin D. D. Li S. S. Shu Q. Y. Gu Z. Y. Wu Q. Feng C. Y. (2018). Identification of microRNAs and long non-coding RNAs involved in fatty acid biosynthesis in tree peony seeds. *Gene* 666 72–82. 10.1016/j.gene.2018.05.011 29738839

[B138] Yin Y. Jiang X. Ren M. Xue M. Nan D. Wang Z. (2018). AmDREB2C, from *Ammopiptanthus mongolicus*, enhances abiotic stress tolerance and regulates fatty acid composition in transgenic *Arabidopsis*. *Plant Physiol. Biochem.* 130 517–528. 10.1016/j.plaphy.2018.08.002 30096686

[B139] Yuan F. Lyu M. J. A. Leng B. Y. Zheng G. Y. Feng Z. T. Li P. H. (2015). Comparative transcriptome analysis of developmental stages of the *Limonium bicolor* leaf generates insights into salt gland differentiation. *Plant Cell Environ.* 38 1637–1657. 10.1111/pce.12514 25651944

[B140] Zhai Z. Keereetaweep J. Liu H. Feil R. Lunn J. E. Shanklin J. (2018). Trehalose 6-phosphate positively regulates fatty acid synthesis by stabilizing WRINKLED1. *Plant Cell* 30 2616–2627. 10.1105/tpc.18.00521 30249634 PMC6241258

[B141] Zhang J. T. Zhu J. Q. Zhu Q. Liu H. Gao X. S. Zhang H. X. (2009). Fatty acid desaturase-6 (Fad6) is required for salt tolerance in *Arabidopsis thaliana*. *Biochem. Biophys. Res. Commun.* 390 469–474. 10.1016/j.bbrc.2009.09.095 19799856

[B142] Zhou J. J. Liu Q. Q. Zhang F. Wang Y. Y. Zhang S. Y. Cheng H. M. (2014). Overexpression of *OsPIL15*, a phytochrome-interacting factor-like protein gene, represses etiolated seedling growth in rice. *J. Integr. Plant Biol.* 56 373–387. 10.1111/jipb.12137 24279300

